# *MicroRNA-17-92*, a Direct *Ap-2α* Transcriptional Target, Modulates T-Box Factor Activity in Orofacial Clefting

**DOI:** 10.1371/journal.pgen.1003785

**Published:** 2013-09-19

**Authors:** Jun Wang, Yan Bai, Hong Li, Stephanie B. Greene, Elzbieta Klysik, Wei Yu, Robert J. Schwartz, Trevor J. Williams, James F. Martin

**Affiliations:** 1 Department of Molecular Physiology and Biophysics, Baylor College of Medicine, Houston, Texas, United States of America; 2 Institute of Biosciences and Technology, Texas A&M Health Science Center, Houston, Texas, United States of America; 3 Department of Craniofacial Biology, UC Denver, Anschutz Medical Campus, Aurora, Colorado, United States of America; 4 Department of Biology and Biochemistry, University of Houston, Houston, Texas, United States of America; 5 Texas Heart Institute, Houston, Texas, United States of America; 6 Program in Developmental Biology, Baylor College of Medicine, Houston, Texas, United States of America; McGill University, Canada

## Abstract

Among the most common human congenital anomalies, cleft lip and palate (CL/P) affects up to 1 in 700 live births. MicroRNA (miR)s are small, non-coding RNAs that repress gene expression post-transcriptionally. The *miR-17-92* cluster encodes six miRs that have been implicated in human cancers and heart development. We discovered that *miR-17-92* mutant embryos had severe craniofacial phenotypes, including incompletely penetrant CL/P and mandibular hypoplasia. Embryos that were compound mutant for *miR-17-92* and the related *miR-106b-25* cluster had completely penetrant CL/P. Expression of *Tbx1* and *Tbx3*, the DiGeorge/velo-cardio-facial (DGS) and Ulnar-mammary syndrome (UMS) disease genes, was expanded in *miR-17-92* mutant craniofacial structures. Both *Tbx1* and *Tbx3* had functional miR seed sequences that mediated gene repression. Analysis of *miR-17-92* regulatory regions uncovered conserved and functional *AP-2α* recognition elements that directed *miR-17-92* expression. Together, our data indicate that *miR-17-92* modulates expression of critical T-box transcriptional regulators during midface development and is itself a target of Bmp-signaling and the craniofacial pioneer factor *AP-2α*. Our data are the first genetic evidence that an individual miR or miR cluster is functionally important in mammalian CL/P.

## Author Summary

CL/P are very common birth defects in humans. The genetic mechanism underlying CL/P pathogenesis is poorly understood. MiRs, small non-coding RNAs that function to post-transcriptionally regulate gene expression, have been identified as pivotal modulators of various developmental events and diseases. To date, there is no individual miR or miR cluster that has been identified as functionally essential in mammalian CL/P. Here, we have discovered that deletion of *miR-17-92* cluster in mice results in craniofacial malformations including CL/P. Importantly, *MIR-17-92* is located on a critical human chromosome region associated with 13q deletion syndrome, a chromosomal disorder that presents with defects including CL/P, suggesting the advantages of our animal model to study human disease. Moreover, our work demonstrated that *miR-17-92* cluster directly repressed T-box factors, which have critical functions during craniofacial development. We further showed that *miR-17-92* was directly activated by Bmp-signaling and transcription factor AP-2α. Together, our work identified a novel miR-mediated transcriptional network underlying CL/P, providing new insights into craniofacial developmental biology.

## Introduction

The evidence that there is a genetic component underlying CL/P is compelling. Analysis of a Danish cohort of CL/P cases revealed that relatives of patients with CL/P have a higher relative risk for CL/P compared to background risk levels. This notion of CL/P heritability is also supported by twin studies [1], [2]. Genome wide association studies (GWAS) and mouse genetics studies have also pointed to genes and genomic regions that are associated with CL/P [3], [4].

MiRs repress gene expression post-transcriptionally by Watson-Crick base pairing to the seed sites in the 3′UTR of target genes. The *miR-17-92* cluster, encoding *miR-17*, *miR-18a*, *miR-19a*, *miR-20a*, *miR-19b-1*, and *miR-92a-1*, is within a region on chromosome 13q that when deletion is associated with CL/P, lung hypoplasia, microphthalmia, microcephaly, and small stature in human patients and has phenotypic similarities to Feingold syndrome [5], [6]. Moreover, *miR-17-92* is found in an amplified region associated with small cell lung cancer, as well as in B-cell lymphomas, and is over-expressed in several solid tumor types, including breast, colon, lung, pancreas, and prostate cancers [7].

The mouse embryos with *miR-17-92* loss-of-function have smaller body size, microphthalmia, heart defects, and lung hypoplasia [6], [8], [9]. Moreover, the *miR-17-92* gain-of-function mutants develop lymphoma, indicating that the mouse is an accurate model for the human syndrome [10]. Unlike the *miR-17-92* loss-of-function mice, its two homologous clusters, *miR-106a-363* and *miR-106b-25* loss-of-function embryos do not exhibit any gross abnormalities.

Our previous findings indicated that *miR-17-92* is directly regulated by Bmp-signaling in heart development [9]. Bmp-signaling deficiency in mice and humans has been shown to cause CL/P and other craniofacial anomalies [11], [12]. Interestingly, *miR-17-92* has also been shown to be directly regulated by Myc family transcription factors [7]. Here, we show that *miR-17-92* deficiency results in orofacial clefting and that the human disease genes *Tbx1* and *Tbx3* are direct targets for *miR-17-92*. Our findings also reveal that *miR-17-92* is a direct target for the master regulator of cranial neural crest development *AP-2α*.

## Results

### *miR-17-92* mutant embryos have orofacial clefting

**Figure 1:**
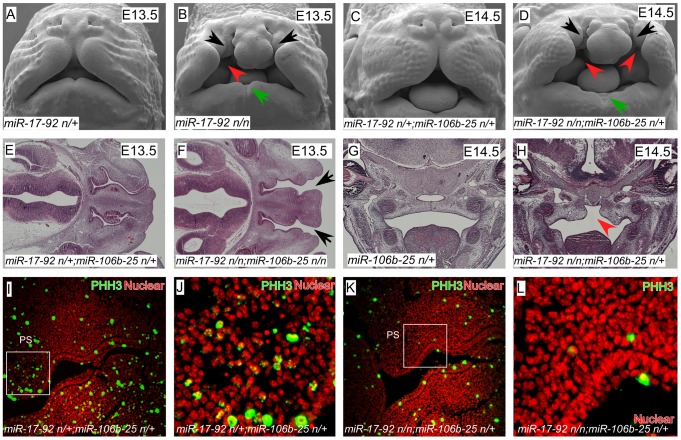
*miR-17-92* cluster is required for midface and palate morphogenesis. (A–D) Scanning electron microscopy (SEM) of embryos with the designated genotypes and designated stages (frontal views). *miR-17-92* mutants and *miR-17-92*; *miR-106b-25* compound mutants exhibit broad orofacial morphogenesis defects including cleft lip (black arrows), mandibular hypoplasia (green arrows), and cleft palate (red arrowheads). (E–H) Histologic analysis with Hematoxylin-eosin (HE) staining of embryos with labeled genotypes and stages. Transverse sections of E13.5 mouse embryos (E, F) and coronal sections of E14.5 mouse embryos (G, H). Black arrows designate cleft lip and red arrowheads designate cleft palate. (I–L) Immunofluorescence (coronal sections) shows proliferation defects in E12.5 mutant embryos: proliferating cells (green)-stained with Phospho-Histon3 (PHH3); Nuclei (red)-stained with DAPI.

We found that *miR-17-92* (*miR17-92^null/null^*) mutant embryos had severe craniofacial defects including CL/P and mandibular hypoplasia with notching, revealing that *miR-17-92* is a critical regulator of craniofacial development (Figure 1A–H, Figure S1). Moreover, by genetically reducing *miR-106b-25* dose on the *miR-17-92^null^* background, the clefting phenotype was both more severe and completely penetrant, indicating that there is genetic redundancy between these two miR complexes (Figure 1C–F, Figure S1E–H and Table S1).

In addition to cleft lip and mandible defects, both *miR-17-92* mutants and *miR-17-92^null^*; *miR-106b-25^null^* compound mutants had cleft secondary palate (Figure 1 B, D, H). Expression of mitotic cell marker phospho-Histone H3 (pHH3) was greatly reduced in *miR-17-92* mutants, indicating that *miR-17-92* is required for normal progenitor cell proliferation during orofacial development (Figure 1I–L, Figure S2). Taken together, these data provide the first genetic evidence that miRs are important regulators of mammalian orofacial development and are involved in CL/P.

### *miR-17-92* is expressed in craniofacial structures

**Figure 2:**
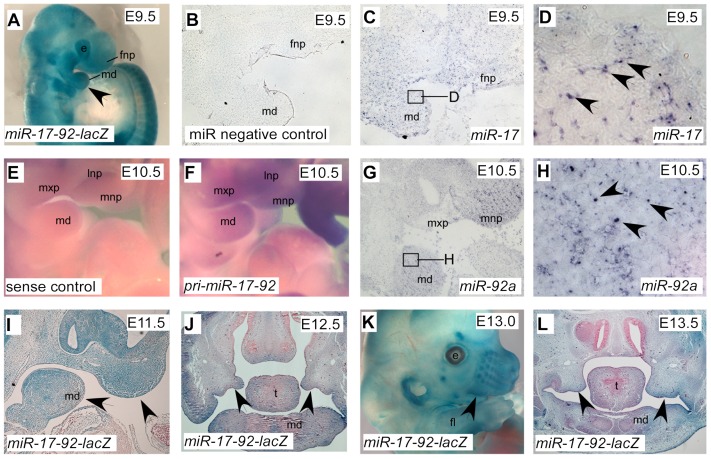
Expression pattern of *miR-17-92* at different embryonic stages. β-Gal stained *miR-17-92-lacZ* BAC transgenic whole embryo (A), embryonic head (K) and craniofacial sections (I, J, L) with the designated stages. Arrowheads indicate positive structures. *In situ* analysis of *pri-miR-17-92* (E, F) and mature *miR-17* and *miR-92a* (B–D, G–H) at facial area. Boxed areas in C and G are correspondingly shown at higher magnification in D and H. e, eye; fl, fore-limb; fnp, frontonasal process; md, mandibular process; mnp, medial nasal process; mxp, maxillary process; lnp, lateral nasal process; ns, nose; t, tongue.

We generated a *miR-17-92* bacterial artificial chromosome (BAC) transgenic LacZ reporter line to follow the expression of primary *(pri)-miR-17-92* (Figure S3A). Three individual transgenic lines showed similar LacZ expression pattern, revealing that *pri-miR-17-92* was expressed in branchial arches and frontonasal process (Figure 2A). LacZ was also detected in the nasal structures, calvarial bones, auricle, periocular mesenchyme, and limb mesenchyme (Figure 2K). Sagittal sections on E11.5 embryos revealed LacZ activity in epithelium and mesenchyme of first branchial arch and frontonasal process (Figure 2I). Coronal sections through E12.5 and E13.5 embryos demonstrated LacZ staining in distal tips of the palatal shelves, the mandibular mesenchyme and mesenchyme of forming frontal bones (Figure 2J, L).

*In situ* with a *pri-miR-17-92* probe revealed similar expression pattern as the transgenic LacZ data (Figure 2E–F). Furthermore, *in situ* analysis with locked nuclei acid (LNA) probes to detect mature *miR-17* and *miR-92a* showed that *miR-17* and *miR-92a* were highly expressed in branchial arch and frontonasal process (Figure 2 B–D, G–H). Unlike *miR-17-92*, expression of *miR-106b-25* was relatively low (Figure S3B–E).

### Tbx genes are repressed by *miR-17-92*

**Figure 3:**
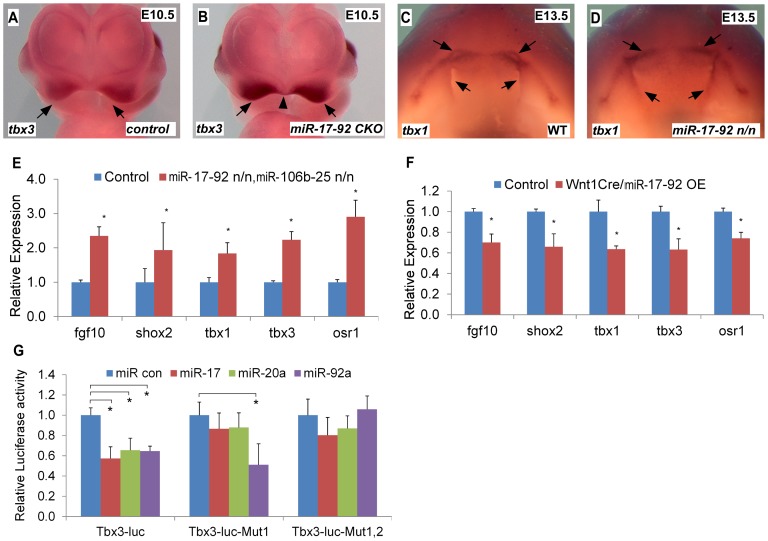
*miR-17-92* represses genes important for craniofacial development. (A–D) Whole mount *in situ* hybridization with indicated probes in mouse embryos with designated genotypes. Black arrows and arrowhead designate expression pattern. (E) qRT-PCR data indicate that expression of *Fgf10*, *Shox2*, *Tbx1*, *Tbx3* and *Osr1* is elevated in *miR-17-92*; *miR-106b-25* compound knock out mutants. (F) Overexpression of *miR-17-92* in cranial neural crest results in repression of *Fgf10*, *Shox2*, *Tbx1*, *Tbx3* and *Osr1*. (G) Luciferase reporter assays with *Tbx3* reporters and miRs as labeled (See Fig. S5 for miR seed sites and mutations). mean ±s.e.m., * indicates statistically significant difference, Student's *t*-test (P<0.05).

*Tbx1* gain-of-function causes cleft lip and is a *miR-17-92* target in the heart [9], [13]. We evaluated the expression of candidate craniofacial *miR-17-92* target genes based on bioinformatics analysis, including *Tbx3*, *Fgf10*, *Pax9*, *Shox2* and *Osr1*, in both *miR-17-92* null and conditional knock out mutants using *AP-2α ^cre^* driver [14]. *In situ* hybridization in *miR-17-92* mutants demonstrated up-regulated *Tbx3* expression in mandible, frontonasal-derived structures, tongue, and secondary palate at E13.5 (Figure S4A–F, Figure S5E–F) and in paired maxillary processes and nasal process at E10.0 and E10.5 (Figure 3A–B, Figure S4 G–N). Changes in *Tbx1* and *Fgf10* expression were not detected at E10.0 likely because the expression changes were not dramatic enough to be detected by *in situ* hybridization (data not shown). *Tbx1* was expanded primarily in the secondary palate, tongue, and oral ectoderm at E13.5 (Figure 3C–D, Figure S5A–D). In addition, *Fgf10* was expanded in distal mandible and tongue (Figure S5I–L), while ectopic *Shox2* expression was observed in distal mandible at E13.5 (Figure S5G–H). Expression of *Osr1* was upregulated in the distal mandible and frontonasal structures at E13.5 (Figure S5M–P). In contrast, the expression pattern of *Pax9* in *miR-17-92* null mutant embryos was unchanged compared to control embryos (data not shown). qRT-PCR experiments also showed up-regulation of *Tbx1*, *Tbx3*, *Fgf10*, *Shox2* and *Osr1* in *miR-17-92*; *miR106b-25* compound mutants at E13.5 (Figure 3E).

To evaluate the expression changes of the above genes, we used a *miR-17-92* conditional, cre-activated gain-of-function line (*miR-17-92^OE^*) and the *Wnt1^cre^* driver to activate *miR-17-92* in cranial neural crest (CNC) [10], [15]. qRT-PCR analysis from *Wnt1^cre^*; *miR-17-92^OE^* orofacial tissue revealed that *Fgf10*, *Tbx1*, *Tbx3*, *Osr1* and *Shox2* were significantly repressed (Figure 3F), while there was no obvious morphological defect detected in *miR-17-92* overexpression mutants potentially due to moderaterepression of the *miR-17-92* target genes.

### Direct regulation of craniofacial development genes by *miR-17-92*

Target genes that are repressed by *miR-17-92* have a mixture of *miR-17/20a/106b* and *miR-92a*/25 family seed sites in their 3′UTRs (Figure S6). Bioinformatics analysis revealed conserved *miR-17/20a/106b* family seed sequence in the 3′ UTR of *Fgf10*, *Shox2* and *Osr1* (Figure S6A–C, F). The *Tbx3* 3′ UTR contained both a *miR-17/20a/106b* family seed site and a *miR-92a*/25 family seed site (Figure S6D–E). We cloned the 3′ UTRs of *Fgf10*, *Shox2*, *Tbx3* and *Osr1* into luc reporter plasmids to test miR seed sequence function *in vitro*. Transfections with miR mimics of *miR-17-92* resulted in drastic reduction in luciferase activity for all of the reporter plasmids (Figure 3G, Figure S7). Mutation of the respective miR seed sequences within 3′ UTRs of those genes ablated the inhibition by the corresponding miR (Figure 3G, Figure S7). These data suggest that *miR-17-92* directly inhibits *Fgf10*, *Shox2*, *Tbx3* and *Osr1*.

### Bmps regulate *miR-17-92* complex in craniofacial development and *miR-17-92* overexpression suppresses orofacial clefting in Bmp mutant mice

**Figure 4:**
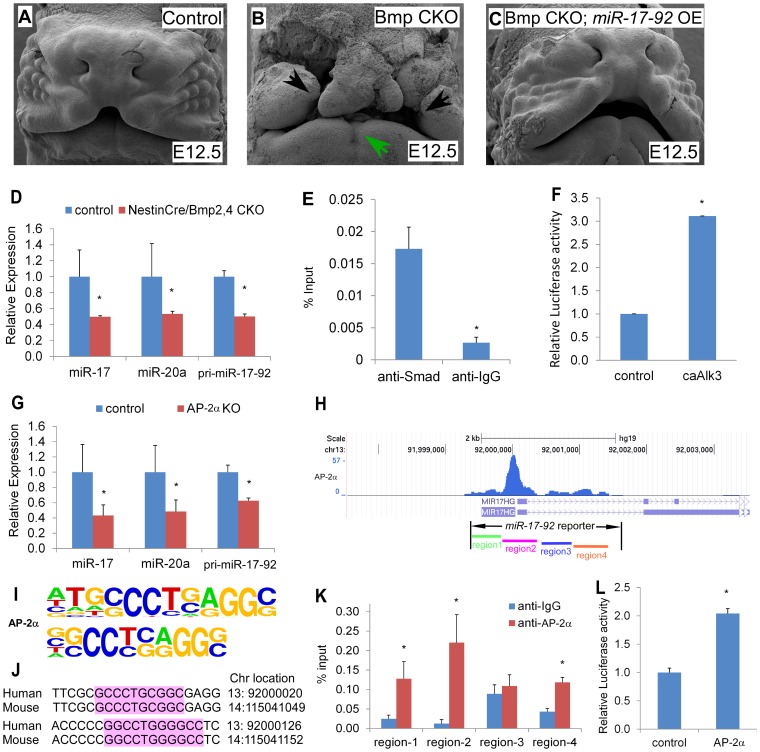
Bmp signals and AP-2α regulate the *miR-17-92* complex in craniofacial structures. (A–C) *miR-17-92* overexpression rescue the orofacial cleft in Bmp mutant mice. Scanning electron microscopy (SEM) of embryos with the designated genotypes and designated stages (frontal views). Broad orofacial morphogenesis defects including severe bi-lateral cleft (black arrows) and mandibular hypoplasia (green arrows) are observed in a *Nestin^Cre^*, *Bmp4 ^flox/flox^*, *Bmp7 ^flox^* ^/+^ (*Bmp* CKO) mutant embryo (B) while not in a control (A) and a *Nestin^Cre^*, *Bmp4 ^flox/flox^*, *Bmp7 ^flox^* ^/+^, *miR-17-92^OE^* embryos (*Bmp* CKO, *miR-17-92* OE) (C). (D–G) Bmp signals directly regulate *miR17-92*. (D) qRT-PCR data indicate that *miR-17-92* was reduced in Bmp mutants. (E) *In vivo* Smad-ChIP-qPCR analysis of the regulatory region in the *miR17-92* promoter. (F) *miR-17-92* upstream Luc reporter activity co-transfected with constitutively active Alk3 (caAlk3). (G–L) AP-2α directly regulates *miR-17-92*. (G) qRT-PCR data indicate that *mir-17-92* is reduced in AP-2α mutants. (H) ChIP-seq peaks indicate AP-2α binding regions in *miR-17-92* chromatin. (I) AP-2α binding sites information based on overrepresented motifs enriched by ChIP-Seq of neural crest cells [16]. (J) Sequence alignment of two predicted AP-2α binding sites in region-2 of *miR-17-92*. (K) *In vivo* AP-2α ChIP-qPCR analysis. (L) Co-transfection data of *miR-17-92* Luc reporter and AP-2α. mean±s.e.m., * indicates statistically significant difference, Student's *t*-test (P<0.05).

Previous work showed that conditional inactivation of *Bmpr1a*, *Bmp4*, and *Bmp2*;*Bmp4* in developing facial processes using the *Nestin^cre^* transgenic driver result in orofacial clefting ([11] and Figure S8). This cre driver directs cre activity in facial prominences [11]. Moreover, *miR-17-92* is a direct target for Bmp-signaling in cardiac progenitors [9]. We crossed the *miR-17-92^OE^* line into *Nestin^cre^*, *Bmp4*, *Bmp7* conditional mutant background to test whether *miR-17-92* gain-of-function could genetically rescue the defects in *Bmp* mutants. All *Nestin^Cre^*, *Bmp4 ^flox/+^*, *Bmp7 ^flox^* ^/+^ embryos (23 out of 23) and embryos without *Nestin^Cre^* (29 out of 29) had normal morphology (Figure 4A, Figure S9A, D, table S2), while all *Nestin^Cre^*, *Bmp4 ^flox/flox^*, *Bmp7 ^flox^* ^/+^ mutant embryos (6 out of 6) had bi-lateral cleft lip and heart defects with incompletely penetrant embryonic lethality at E12.0 likely due to heart defects (Figure 4B, Figure S9B, E, table S2). Most (5 out of 6) *Nestin^Cre^*, *Bmp4 ^flox/flox^*, *Bmp7 ^flox^* ^/+^, *miR-17-92^OE^* embryos were rescued by *miR-17-92* overexpression (significant different compared to *Nestin^Cre^*, *Bmp4 ^flox/flox^*, *Bmp7 ^flox^* ^/+^ mutants, CHI-TEST, p<0.01), with full suppression of cleft lip and heart defect caused by Bmp loss-of-function, but not eye defect (Figure 4C, Figure S9C, F, table S2).

Consistently, qRT-PCR data indicated that *pri-miR-17-92*, *miR-17*, and *miR-20a* were reduced in *Bmp2/4* mutant midface (Figure 4D). *In situ* analysis using *miR-17* LNA probe also indicated that *miR-17* was dramatically reduced in *Bmp2/4* mutants (Figure S10C, D) compared to controls (Figure S10A, B). In addition, qRT-PCR indicated that *Fgf10*, *Tbx1*, *Tbx3*, *Osr1* and *Shox2* were up-regulated in the midface of *Bmp2*; *Bmp4* conditional mutants (Figure S10E), further suggesting that these genes are regulated by a BMP-*miR-17-92* genetic pathway in craniofacial structures.

Moreover, *in vivo* chromatin immunoprecipitation (ChIP) data using embryonic midface extracts showed enrichment in the anti-Smad1/5/8 immunoprecipitated chromatin, indicating that Smad1/5/8 directly binds *miR-17-92* chromatin (Figure 4E). Co-transfection of a constitutively active Bmpr1a construct with *miR-17-92* luc reporter resulted in approximately 3-fold induction supporting the hypothesis that Bmp signaling directly regulates *miR-17-92* in developing craniofacial structures (Figure 4F). Together, a conserved Bmp-*miR-17-92* genetic pathway plays a critical role in the orofacial development.

### *AP-2α* regulates *miR-17-92* in craniofacial development

Mouse mutants for *AP-2α* have CL/P and human patients have branchio-oculo-facial syndrome that has CL/P as a cardinal feature (BOFS MIM 113620). ChIP-sequencing (ChIP-seq) indicated that *AP-2α* bound to *miR-17-92* chromatin in cultured human neural crest [16] (Figure 4H, S11A). To determine if *AP-2α* directly regulates *miR-17-92*, we evaluated *pri-miR-17-92*, mature *miR-17*, and mature *miR-20a* levels in the *AP-2α* mutant midface. qRT-PCR experiments indicated that *pri-miR-17-92* and mature miRs were down-regulated in *AP-2α* mutants (Figure 4G). We used ChIP-PCR to determine whether *AP-2α* binds to *miR-17-92* chromatin in developing midface tissue. Because there are multiple predicted *AP-2α* binding sites in *miR-17-92*, we subdivided *miR-17-92* into four regions based on ChIP-seq (Figure 4 H–J and S11). ChIP-PCR experiments using midface extracts indicated that *AP-2α* bound to *miR-17-92* regions 1, region 2, and region 4 (Figure 4 K). Transfection experiments with a *miR-17-92* reporter containing *AP-2α* binding sites revealed that *AP-2α* transcriptionally activated *miR-17-92* although synergism with Smad1 was not detected using this *miR-17-92* reporter (Figure 4 L). Moreover, *AP-2α* may also directly regulate *miR-106b-25* as suggested by the analysis of ChIP-seq data [16] (Figure S12).

## Discussion

We report the first miR underlying mammalian CL/P. *miR-17-92* is located on human chromosome 13q31.3 in a critical region for CL/P associated with 13q deletion syndrome highlighting the importance of our findings to human disease. Our data indicated that *miR-17-92* promotes proliferation in developing midface by regulating a group of progenitor genes including *Tbx1* and *Tbx3* that are known human disease genes (Figure S13). Our findings reveal that timely down-regulation of progenitor genes in developing midface by *miR-17-92* is critical for normal midface development.

### *Mir-17-92* regulates *Tbx1* and *Tbx3* genes in craniofacial development

*Tbx1* loss- and gain-of-function result in cleft palate in human DGS patients and mouse models [13], [17]–[19]. Consistent with our findings, *Tbx1* gain-of-function results in cell cycle arrest [17]. DGS is characterized by highly variable phenotypes indicating that there are strong modifiers in the human genome [18], [20]. Our findings suggest *miR-17-92* as a candidate genetic modifier for *Tbx1* since it fine-tunes *Tbx1* expression levels.

Mouse mutants for *Tbx3* and the related *Tbx2* have cleft palate [21]. Furthermore, human patients with UMS have abnormal and distinct facial appearance indicating a requirement for *Tbx3* in human craniofacial development [22]. While our findings suggest that elevated *Tbx3* inhibits proliferation, there is other evidence suggesting that *Tbx3* promotes proliferation [23]. However, an *in vivo* study reveals that *Tbx3* overexpression results in reduced cardiomyocyte proliferation in the zebrafish heart [24]. More work will be required to evaluate *Tbx3* function and target genes *in vivo* in the context of the *miR-17-92* mutant midface to better understand contextual *Tbx3* function.

### *MiR-17-92* regulates Fgf signaling

Both *Fgf10* and *Fgfr* null mice have cleft secondary palate [25], [26]. Mutations in *Fgf10* and Fgf receptors cause lacrimo-auricular-dento-digital (LADD) syndrome in human patients indicating a requirement for Fgf-signaling in human craniofacial development [27]. *Fgf10* mRNA is enriched in anterior and middle regions of the secondary palate. Moreover, *Fgf10* deficiency results in abnormal fusion of the palatal to oral cavity epithelium, suggesting that *Fgf10* is required for maturation of palate epithelium. Importantly, elevated Fgf signaling is pathologic in human patients as shown by the extensive investigations into Fgf receptor mediated craniosynostosis [28]. Homozygosity for the *Fgfr2* gain-of-function Crouzon mutation in mice results in cleft palate, as well as, craniosynostosis [29] indicating that elevated Fgf signaling also causes cleft palate. Our data demonstrate that *miR-17-92* directly represses *Fgf10* as a mechanism to maintain correct levels of *Fgf10* during palate closure.

### Micro RNAs in human orofacial development and disease

Currently, there are no other genetic loss-of-function data indicating that single miRs or miR clusters are important in mammalian orofacial clefting. Data from zebrafish indicate that *miR-140* targets *pdgfra* to regulate primary palate development [30]. GWAS in human patients reveal important genome regions that are associated with CL/P, including 8q24 [3], [31]. Within the 8q24 region is the c-myc gene, a known *miR-17-92* regulator [32], [33]. Chromosomal deletions that include *miR-17-92* cause a variant of Feingold syndrome in human patients with small stature and skeletal abnormalities [6]. Human patients with hemizygous *miR-17-92* deletion do not have CL/P likely reflecting phenotypic heterogeneity in *miR-17-92* loss of function families. These data are consistent with our findings indicating that there is incomplete penetrance of the CL/P phenotype in *miR-17-92* mutant mouse embryos (table S1).

### *Mir-17-92* regulation in midface development by Bmp and *AP-2α*

Consistent with previously finding that Bmp-deficiency results in CL/P in mice and humans [11], [12], our data indicate that Bmp signaling activates *miR-17-92* in craniofacial development. Moreover, we show that *AP-2α* also regulates miR-17-92 expression although our transfection assays failed to uncover synergistic *miR-17-92* activation by *AP-2α* and Bmp-signaling (not shown). One possibility is that Bmp-signaling and *AP-2α* activate *miR-17-92* sequentially during craniofacial progenitor cell development. The assays we employed here cannot easily distinguish molecular events that occur in neighboring or closely apposed cells rather than in the same cell. We also failed to detect up-regulated *miR-17-92* target genes in *AP-2α* mutants perhaps due to functional redundancy with other *AP-2* family members [34]–[36]. Nonetheless our findings have important implications since *AP-2α* has been shown to regulate *Irf6*, a common genetic defect in syndromic and non-syndromic CL/P in human patients [37]. *AP-2α* regulation potentially connects *miR-17-92* to a gene regulatory network that may be involved in a large portion of human CL/P. In summary, we identified a miR-mediated genetic pathway that plays critical roles during orofacial development (Figure S13).

## Materials and Methods

### Ethics statement

All animal experiments detailed within the manuscript were approved by the Baylor College of Medicine review board.

### Mouse alleles and transgenic lines

The *miR-17-92* and *miR-106b-25* alleles, *Bmp2*, *Bmp4* and *Bmp7* conditional null, *AP-2α ^cre^*, *Nestin ^cre^* and *Wnt1^cre^* alleles were previously described [8], [10], [11], [14], [15], [38]. To generate *miR-17-92-lacZ* reporter transgenic lines, we obtained the BAC from BACPAC Resources Center, Children's hospital Oakland Research Institute (BAC number: RP23-89P9) and replaced *miR-17-92* sequence with lacZ coding sequence by recombineering, followed by pro-nuclear injection. Constructs were generated using PCR, cloning and recombineering. A LoxP site flanked neo cassette was isolated from PL452 plasmid using BamHI and EcoRI. *lacZ* coding sequence was isolated from hsp68-lacZ plasmid using BamHI and NcoI. All fragments were cloned into pBluescript SK+ to generate *miR-17-92-lacZ* construct, followed by recombineering and subsequently Cre mediated recombination for removal of the neo cassette (Figure S3A).

### Scanning Electron Microscopy (SEM)

Mouse embryos were harvested in ice-cold Phosphate Buffered Saline (PBS), then fixed overnight (O/N) in 4% paraformaldehyde (PFA) and 2% glutaraldehyde in PBS at 4°C. The samples were then dehydrated in ethanol series to a final 100% ethanol, followed by transferring to graded series of increasing concentrations of hexamethyldisilazane (HMDS) for 5 min each and air dried O/N. Samples were mounted on to double-stick carbon tabs (Ted Pella. Inc.), which have been pre-mounted on to aluminum specimen mounts (Electron Microscopy Sciences). The samples were then coated with a thickness of 25 nm platinum alloy under vacuum using a Balzer MED 010 evaporator (Technotrade International), then immediately flash carbon coated under the same vacuum. The samples were transferred to a desiccator for later examination. JSM-5910 scanning electron microscope (JEOL, USA, Inc.) was used at an accelerating voltage of 5 kV.

### Immunofluorescence

Embryos were fixed in 4% PFA, embedded in paraffin and cut to 5 µm sections mounted on Superfrost/Plus slides (Fisher Scientific). The antigens were retrieved by incubating in the citrate buffer (10 mM) for 2 minutes in microwave oven. The primary antibody was anti-Phospho-Histone H3 with 1∶200 dilution (Cell Signaling). Broad HRP conjugated secondary antibody (Invitrogen) was used and visualized using TSA Plus Fluorescence Systems from PerkinElmer on a Zeiss LSM 510 Confocal Microscope. Nuclei were stained with 4,6-diamidino-2-phenylindole (DAPI).

### *In situ* hybridization

Tissue preparation and *in situ* hybridization were as previously described [39], [40]. The gene probes were synthesized using DIG RNA Labeling Kit (Roche) following manufacturer's guidelines. The enzymes used for digestion and transcription of *in situ* constructs are SacII and T7 for *Fgf10*, XhoI and T7 for *Shox2* (gift from Dr. Yiping Chen's lab), EcoRI and T7 for *Osr1*(gift from Dr. Rulang Jiang's lab), EcoRI and T3 for *Tbx1*(gift from Dr. Antonio Baldini's lab), PstI and T3 for *Tbx3* (gift from Dr. Robert Kelly's lab). miRCURY LNA probes were purchased from Exiqon and used per manufacturer's guidelines.

### Generation of constructs

To generate 3′ UTR luciferase reporter plasmids, 3′ UTR genomic sequence of genes including *Fgf10*, *Osr1*, *Shox2* and *Tbx3* were amplified using a high-fidelity PCR system (Roche) with designed oligonucleotides and subcloned into the pMIR-REPORT Luciferase miRNA Expression Reporter Vector (Ambion).

Oligonucleotides used to amplify 3′ UTR genomic sequence of *Fgf10* are sense, 5′-CGACTAGTAAGAAAACACTGTTGGTGGATGCAG -3′, and antisense, 5′-GCACGCGTTTTTATTCTCTTTTCCCAGC-3′. Oligonucleotides used to amplify 3′ UTR genomic sequence of *Osr1* are sense, 5′- GACTAGTATAAACAGAGCCTGCGGG -3′, and antisense, 5′- CGACGCGTGCCTGTAAAATAACCGTTTATTT -3′. Oligonucleotides used to amplify 3′ UTR genomic sequence of *Shox2* are sense, 5′-ACTAGTCGCCGGCGCCAGCGCCACGGT-3′, and antisense, 5′-AAGCTTCTTTTTTGTATGAACGTCC-3′. Oligonucleotides used to amplify 3′ UTR genomic sequence of *Tbx3* are sense, 5′- GACTAGTAAACAAGAAAAACAAAATCGCC -3′, and antisense, 5′- CCCAAGCTTTCATTTCAATAAAAATTTATTG -3′. Oligonucleotides used to amplify 3′ UTR genomic sequence of *Tbx3* without *mir17/mir-20a* seed site are sense, 5′- GACTAGTGTGTAACCAGGCTGCTGTTGCTTT -3′, and antisense, 5′- CCCAAGCTTTGGTCGTTTGAACCAAGTCCCTCT -3′. Underlined letters represent enzyme restriction sites for subcloning. All PCR products were sequenced to make sure no mutations were introduced.

### Site-directed mutagenesis

All site-directed mutagenesis of the miR seed sites in the 3′ UTR reporter constructs were achieved by using the QuikChange II site-directed mutagenesis kit (Stratagene). The sense-strand sequences of the oligonucleotides used for mutagenesis (underlined letters indicate the mutation of miR seed sites) were: 5′-TAAGACACGCAAGCATTTACTGGAAAGACACTGGGTCATATCATATGCACAACCAAAG- 3′ (*Fgf10*-mut1,), 5′-CCCCATGCGCTCTCAGTTGACTTAATTTGACACTCTGCAATAAAAAACACCAGCAAT- 3′ (*Fgf10*-mut2), 5′-ACAGCAAATAGTGCAGACGTTGGATTCTTATTTCAACCCGCCATTTAGATTACTAAAGAGA- 3′ (*Fgf10*-mut3); 5′-GCTGACCTTTTTCTGCGAAGTTGAATTCAATAGGAGACATTTGATAAGAG - 3′ (*Shox2*-mut); 5′- GCCGGGCGTTGTATTGCGACTGGGAATTCATGCTGACCATCGGTAACGGAC - 3′ (*Osr1*-mut); 5′- GGACCATTAGTTCTTTTAACTGTATAGAATTCAACAAGGTTTTAAAAGATAATAATA - 3′ (*Tbx3*-mut). All PCR products were sequenced to make sure no unexpected mutations were introduced.

### Chromatin immunoprecipitation

Wild type mouse embryonic orofaces were dissected at E12.5 (for Smad1/5/8 ChIP) or E10.5 (for *AP-2α* ChIP) and followed by ChIP analysis as previously described [9]. As control, normal rabbit immunoglobulin G was used as a replacement for the anti-Smad1/5/8 (sc-6031 X, Santa Cruz) and 3B5 mouse monoclonal *AP-2α* antibody [41] to reveal nonspecific immunoprecipitation of the chromatin. The PCR products were evaluated for appropriate size on a 2% agarose gel and were confirmed by sequencing. The primers for amplifying the regulatory element in the 5′ upstream of *mir-17-92* genomic sequence were: sense, 5′- CTGGCGGGAAGCCTGAGC -3′, antisense, 5′-CACGGCGGCTCGTTCTTG -3′ (for AP-2*α* region1); sense, 5′- CCTTCATTCACCCACATGGTCCTT -3′, antisense, 5′- AGCAGCCGCCACCATCTT -3′ (for AP-2*α* region2); sense, 5′- GCACACAATGGCCCTCGG -3′, antisense, 5′- GCGCGCACAAAGTTTCGG -3′ (for AP-2*α* region3); sense, 5′- CGCAGCCGCCCAGAAAC -3′, and antisense, 5′-TCCGCGCCAGCTTATCAAGAGAAA -3′ (for AP-2*α* region4 and Bmp/Smad regulatory element).

### Real time RT-PCR

Total RNA was isolated using RNeasy Micro Kit (QIAGEN) and real-time thermal cycling was performed using StepOne Real-Time PCR Systems (Applied Biosystems). Super Script II Reverse Transcriptase (Invitrogen) was used for RT-PCR and SYBR Green JumpStart Taq ReadyMix (SIGMA) was used for real-time thermal cycling. All error bars represent SEM.

### Luciferase activity assay

Plasmids used for transfection were generated as described above or previously reported [9]. LS8 cells were transfected using Lipofectamine 2000 (Invitrogen). Luciferase activity assays were measured using the luciferase Assay System (Promega).

### ChIP-seq analysis

hNCC *AP-2α* ChIP-seq and histone modification markers ChIP-seq datasets were accessed from GEO under accession number GSE28876 [16], [42]. Raw fastq reads were mapped to hg19 genome using Bowtie2 [43]. The total number of tags of each ChIP-seq run was normalized to 10 million. ChIP-seq tracks were visualized and compared in UCSC Genome Browser.

## Supporting Information

Figure S1 *miR-17-92* mutant embryos have orofacial clefting. (A–D) *miR-17-92* mutant embryos had bilateral cleft lip or unilateral cleft lip (B, D) and cleft palate (not shown) versus their control littermates (A, C). (E–H) Compared with *miR-17-92* mutant embryos, *miR-17-92* and *miR-106b-25* compound mutants had more severe cleft lip phenotypes and more frequent bilateral cleft lip (F, H) versus their control littermates (E, G).
(TIF)

Figure S2 Proliferation was reduced in *miR-17-92* mutant embryos. (A–H) Immunofluorescence with Phospho-Histon3 (pHH3) antibody (green) at E12.5. Nuclei were stained with DAPI (red). (I–J) Count of pHH3 positive cells in epithelial cells (I) and mesenchymal cells (J) in control and *miR-17-92* mutant embryos. ps, palate shelf; t, tongue.
(TIF)

Figure S3 (A) Schematic diagram of *mir-17-92* bacterial artificial chromosome (BAC) transgenic. The LacZ reporter was introduced into a mouse BAC while concurrently the mature *miR-17-92* sequences from the BAC were removed. (B–E) *In situ* hybridization on craniofacial sections indicated expression of *miR-106b* and *miR-25* in embryonic palate shelf (ps) at E12.5. Black arrows designate signals. Dashed lines show outline of palate shelf.
(TIF)

Figure S4 *mir-17-92* represses *Tbx3*. Whole mount *in situ* hybridization indicated up-regulation of *Tbx3* in *miR-17-92* null (B,D, F,H) and *AP-2α^Cre^* conditional knock-out of *miR-17-92* (J,L, N) mutants compared to controls (A,C, E,G; I, K, M) at designated stages. Black arrows designate signals. Boxed areas in K and L are correspondingly shown at higher magnification in M and N.
(TIF)

Figure S5 *mir-17-92* represses genes important for craniofacial development. Whole mount *in situ* hybridization with indicated probes in mouse embryos with designated genotypes and designated stages. Black arrows designate expressing areas. Embryos were shown in frontal view (C, D, G, H, O, P), lateral view (I, J, M, N) and ventral view of roof of mouth and palatal shelves with lower jaw removed (A, B, E, F, K, L). Black arrows designate signals.
(TIF)

Figure S6 Phylogenetic sequence alignment of miR-17-92 family seed sequence in *Fgf10* 3′ UTR (A and B), *Shox2* 3′ UTR (C), *tbx3* 3′ UTR (D and E) and *Osr1*3′ UTR (F).
(TIF)

Figure S7 *mir-17-92* directly regulates *Shox2*, *fgf10* and *osr1*. (A–C) Luciferase reporter assays with reporters and miRs as labeled. Mean±s.e.m., * indicates statistically significant difference, Student's *t*-test (P<0.05).
(TIF)

Figure S8 Cleft lip and palate in *Bmp* CKO mutants. Genotypes and stages of embryos (A–F) and hematoxylin-eosin (HE) staining sections (G–H) are as labeled. Dashed lines show outline of midface, arrows designate fusion or clefting, arrowheads designate palate.
(TIF)

Figure S9 *miR-17-92* overexpression rescues the orofacial cleft in *Bmp* CKO mutants. Embryos with the designated genotypes and designated stages are shown in side views (A–C) and frontal views (D–F). A *Nestin^Cre^*, *Bmp4 ^flox/flox^*, *Bmp7 ^flox^* ^/+^ (*Bmp* CKO) mutant embryo (B, E) had severe bi-lateral cleft, which was observed in in a control (A, C) and a *Nestin^Cre^*, *Bmp4 ^flox/flox^*, *Bmp7 ^flox^* ^/+^, *miR-17-92^OE^* embryos (*Bmp* CKO, *miR-17-92* OE) (C,F). However, *miR-17-92* overexpression did not rescue the eye defect in a Bmp CKO mutant (C). Black arrows designate and orofacial structures.
(TIF)

Figure S10 *miR-17-92* is a downstream of Bmp signaling. (A–D) *In situ* analysis of mature *miR-17* indicated that *mir-17* was dramatically reduced in a *Nestin^Cre^*, *Bmp4 ^flox/flox^*, *Bmp2 ^flox^* ^/+^ (*Bmp4 −/−*, *Bmp4 +/−* CKO) mutant (C–D) compared to a control (A–B). Boxed areas in A and C are correspondingly shown at higher magnification in B and D. Black arrows designate signals. (E) qRT-PCR data indicate that loss of Bmp signals results in elevation of *miR-17-92* target genes including *Fgf10*, *Shox2*, *Tbx1*, *Tbx3* and *Osr1*. Mean±s.e.m., * indicates statistically significant difference, Student's *t*-test (P<0.05).
(TIF)

Figure S11 AP-2α directly regulates *the miR-17-92* complex. (A) AP-2α and histone modification markers ChIP-seq data in cultured human neural crest and Hela cells. (B) Sequence alignment of AP-2α binding region-1, 3 and 4 in *miR-17-92*. Region 1 contains three potential binding sites, region 3 and 4 contain two. Region two was shown in Figure 4.
(TIF)

Figure S12 *The miR-106b-25* complex is a potential AP-2α target, which is suggested by AP-2α and histone modification markers ChIP-seq data in cultured human neural crest and Hela cells.
(TIF)

Figure S13 A model for the Bmp/AP-2α-*miR-17-92-Tbx* pathway during orofacial development.
(TIF)

Table S1 Summary of phenotypes of *miR-17-92* single and *miR-17-92*;*miR- 106b-25* compound mutant embryos. For cleft lip and palate, the penetrance and severity of the phenotype was more severe in compound mutants for both miR clusters. A “wide mouth” phenotype that was observed in some *miR17-92* mutants represents an increased distance between the two frontonasal processes and we believe this is an intermediate phenotype between normal and cleft lip.
(DOCX)

Table S2 Phenotype summary of embryos with different genotypes. At embryonic day 10.5 to 12.5 (E10.5–E12.5), all *Nestin Cre*, *Bmp4 flox/flox*, *Bmp7 flox*/+ mutant embryos (*Nestin Cre*, *B4 f/f*, *B7f*/+) have severe bi-lateral cleft lip and heart defect. In most case, *miR-17-92* over expression rescue cleft lip and heart defect caused by *Nestin Cre*, *B4 f/f*, *B7f*/+ but not eye defect. 83.33% (5 out of 6) *Nestin Cre*, *Bmp4 flox/flox*, *Bmp7 flox*/+, *miR-17-92-*OE (*Nestin Cre*, *B4 f/f*, *B7f*/+, *miR-*OE) mutant embryo were fully rescued and 16.67% (1 out of 6) had bi-lateral cleft lip and heart defect. Two *Nestin Cre*, *B4 f/f*, *B7f*/+ mutants died at E12.0 likely due to severe heart defect and 7 embryos were not able to genotyped due to early embryonic lethal at E9.5. * *miR-17-92* over expression rescued cleft lip (compared to Bmp CKO, CHI-TEST, p<0.01).
(DOCX)
