# Correction: *MicroRNA-17-92*, a Direct *Ap-2α* Transcriptional Target, Modulates T-Box Factor Activity in Orofacial Clefting

**DOI:** 10.1371/annotation/90602bc3-5052-49ac-a7fb-33210d7c8b4d

**Published:** 2013-12-16

**Authors:** Jun Wang, Yan Bai, Hong Li, Stephanie B. Greene, Elzbieta Klysik, Wei Yu, Robert J. Schwartz, Trevor J. Williams, James F. Martin

The colors in supporting information files Figure S4 and Figure S8 previously did not display correctly and have been replaced with files displaying the correct colors. No other change to the content of the figures has been made.

