# Supplementary figures and images for: MicroRNA-17-92, a Direct Ap-2α Transcriptional Target, Modulates T-Box Factor Activity in Orofacial Clefting
Source: PLoS Genet. 2013 Sep 19;9(9):e1003785. doi: 10.1371/journal.pgen.1003785 (PMC3777996; doi:10.1371/journal.pgen.1003785)

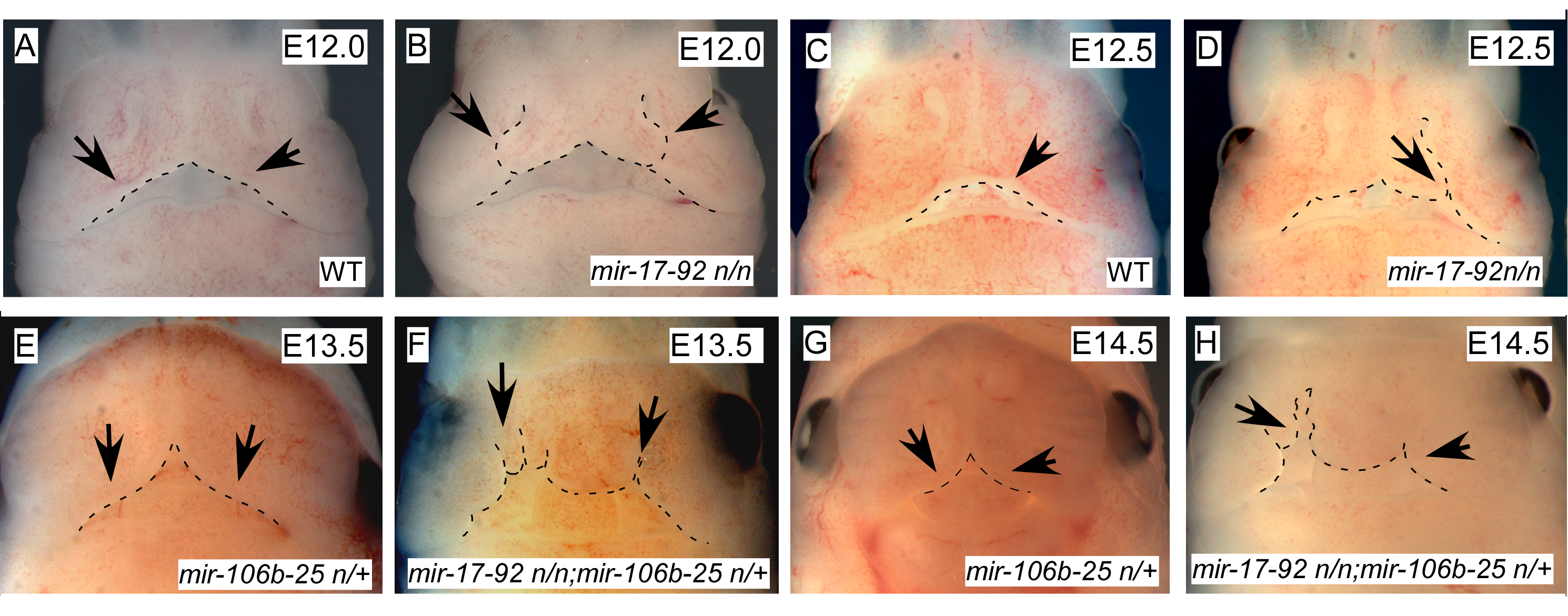

Supplement: Figure S1 — miR-17-92 mutant embryos have orofacial clefting. (A–D) miR-17-92 mutant embryos had bilateral cleft lip or unilateral cleft lip (B, D) and cleft palate (not shown) versus their control littermates (A, C). (E–H) Compared with miR-17-92 mutant embryos, miR-17-92 and miR-106b-25 compound mutants had more severe cleft lip phenotypes and more frequent bilateral cleft lip (F, H) versus their control littermates (E, G). (TIF) [file pgen.1003785.s001.tif]

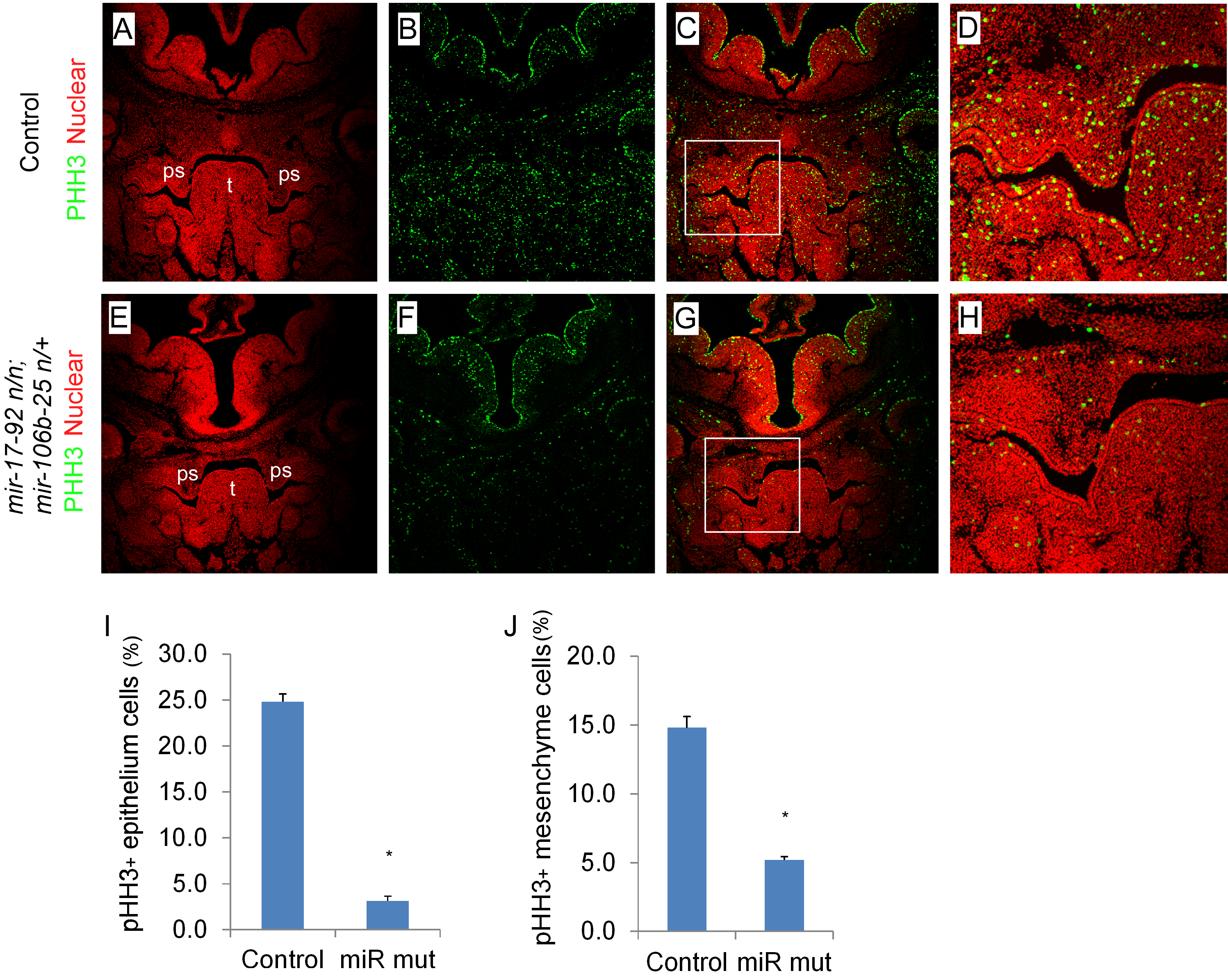

Supplement: Figure S2 — Proliferation was reduced in miR-17-92 mutant embryos. (A–H) Immunofluorescence with Phospho-Histon3 (pHH3) antibody (green) at E12.5. Nuclei were stained with DAPI (red). (I–J) Count of pHH3 positive cells in epithelial cells (I) and mesenchymal cells (J) in control and miR-17-92 mutant embryos. ps, palate shelf; t, tongue. (TIF) [file pgen.1003785.s002.tif]

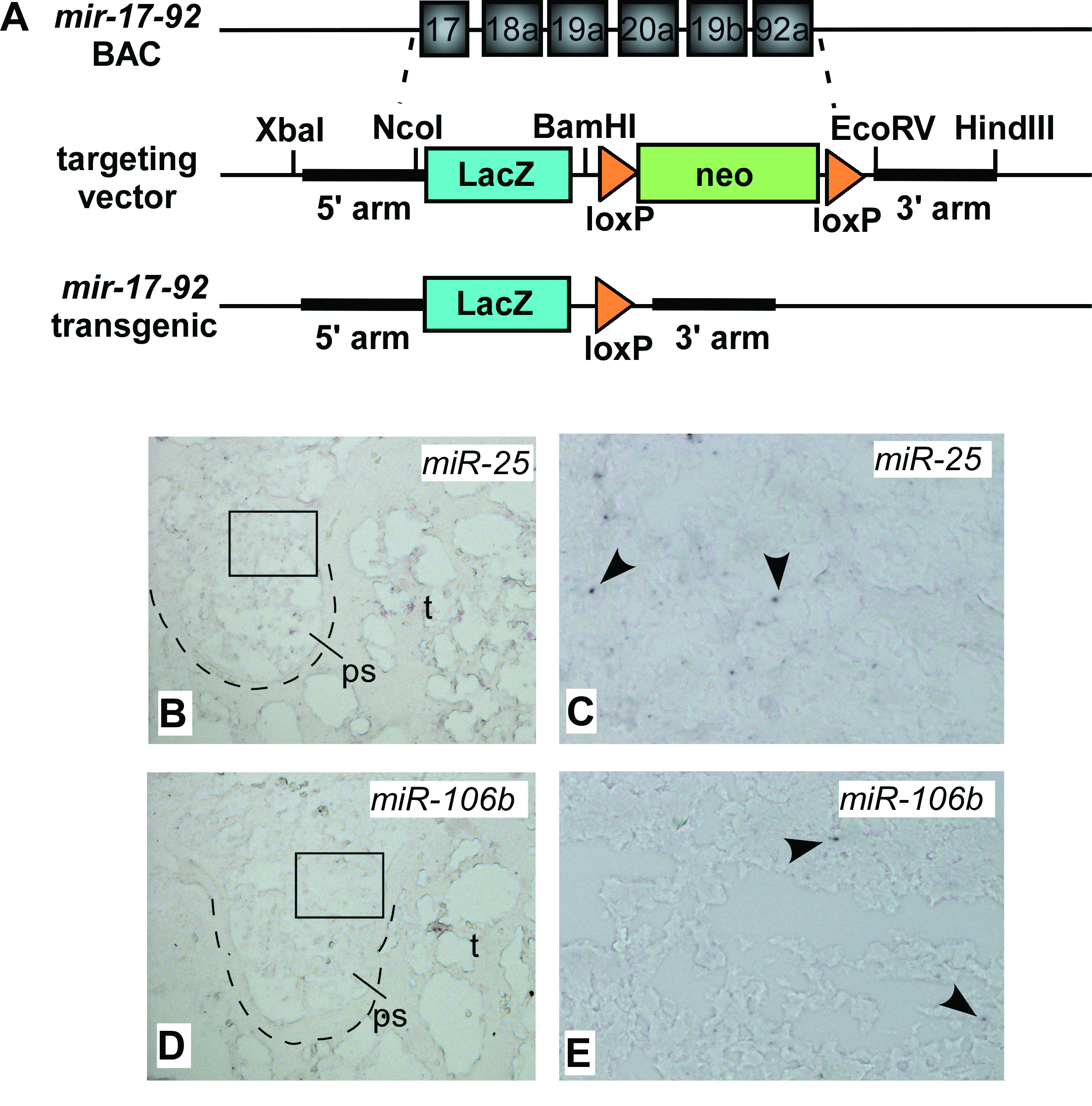

Supplement: Figure S3 — (A) Schematic diagram of mir-17-92 bacterial artificial chromosome (BAC) transgenic. The LacZ reporter was introduced into a mouse BAC while concurrently the mature miR-17-92 sequences from the BAC were removed. (B–E) In situ hybridization on craniofacial sections indicated expression of miR-106b and miR-25 in embryonic palate shelf (ps) at E12.5. Black arrows designate signals. Dashed lines show outline of palate shelf. (TIF) [file pgen.1003785.s003.tif]

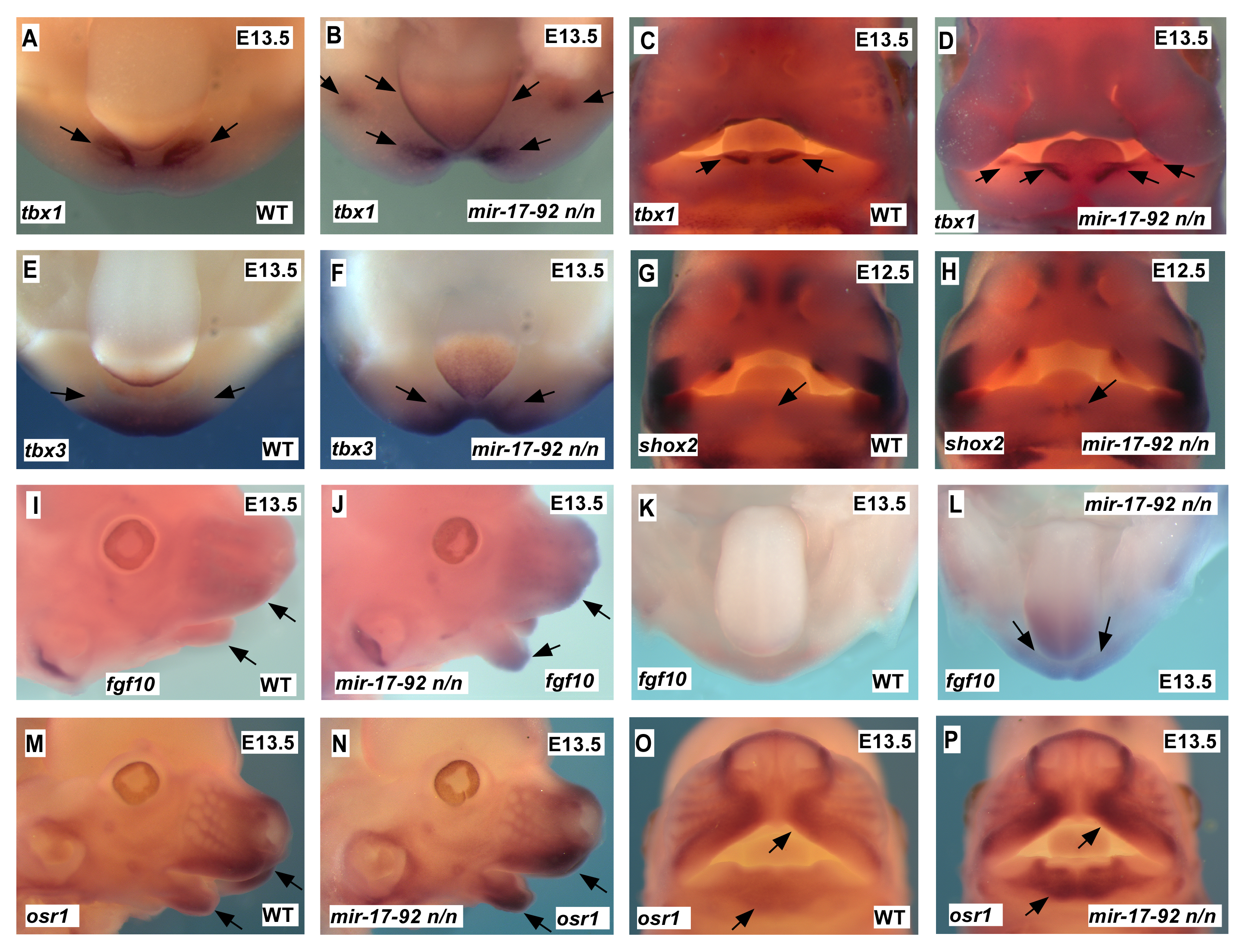

Supplement: Figure S5 — mir-17-92 represses genes important for craniofacial development. Whole mount in situ hybridization with indicated probes in mouse embryos with designated genotypes and designated stages. Black arrows designate expressing areas. Embryos were shown in frontal view (C, D, G, H, O, P), lateral view (I, J, M, N) and ventral view of roof of mouth and palatal shelves with lower jaw removed (A, B, E, F, K, L). Black arrows designate signals. (TIF) [file pgen.1003785.s005.tif]

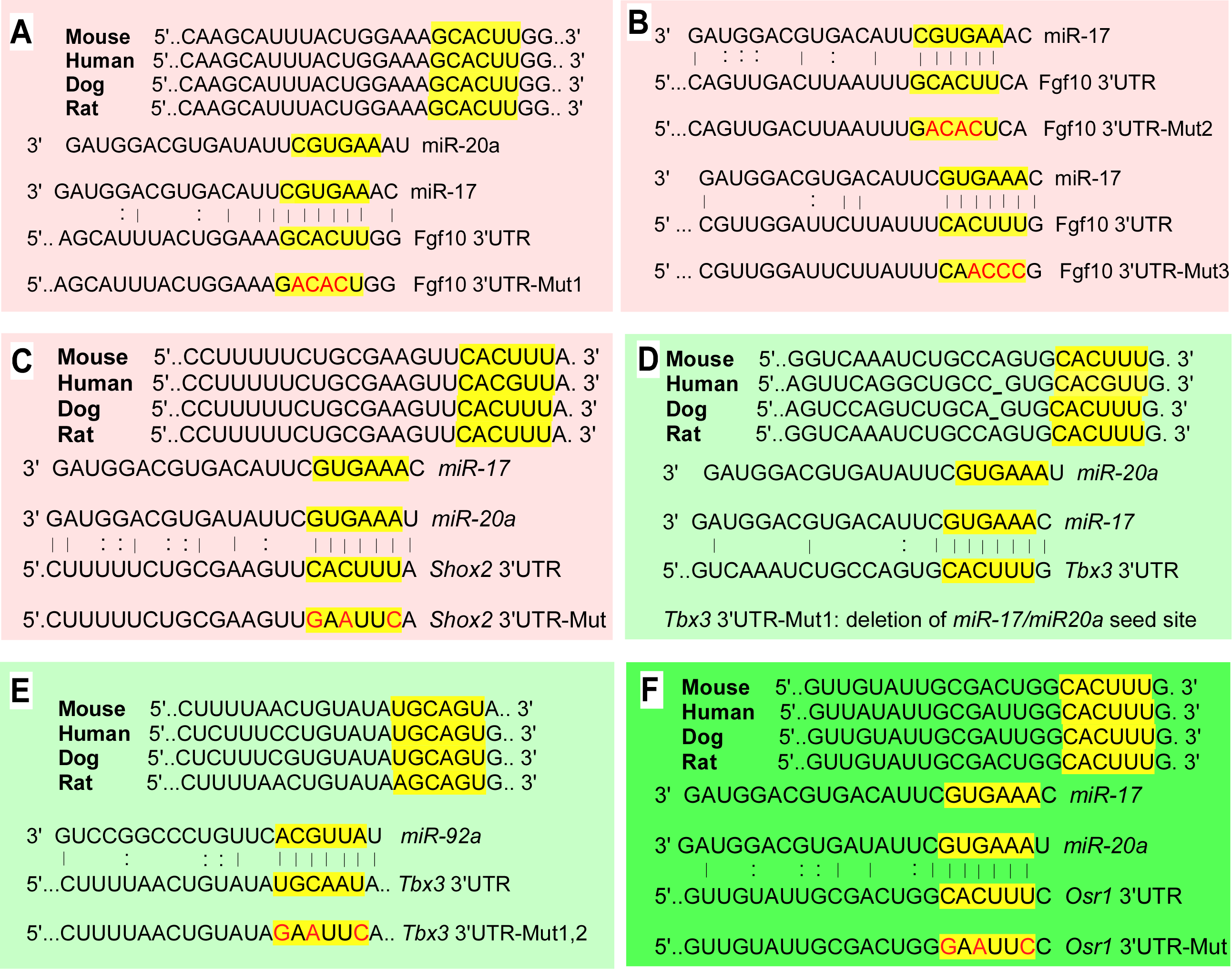

Supplement: Figure S6 — Phylogenetic sequence alignment of miR-17-92 family seed sequence in Fgf10 3′ UTR (A and B), Shox2 3′ UTR (C), tbx3 3′ UTR (D and E) and Osr13′ UTR (F). (TIF) [file pgen.1003785.s006.tif]

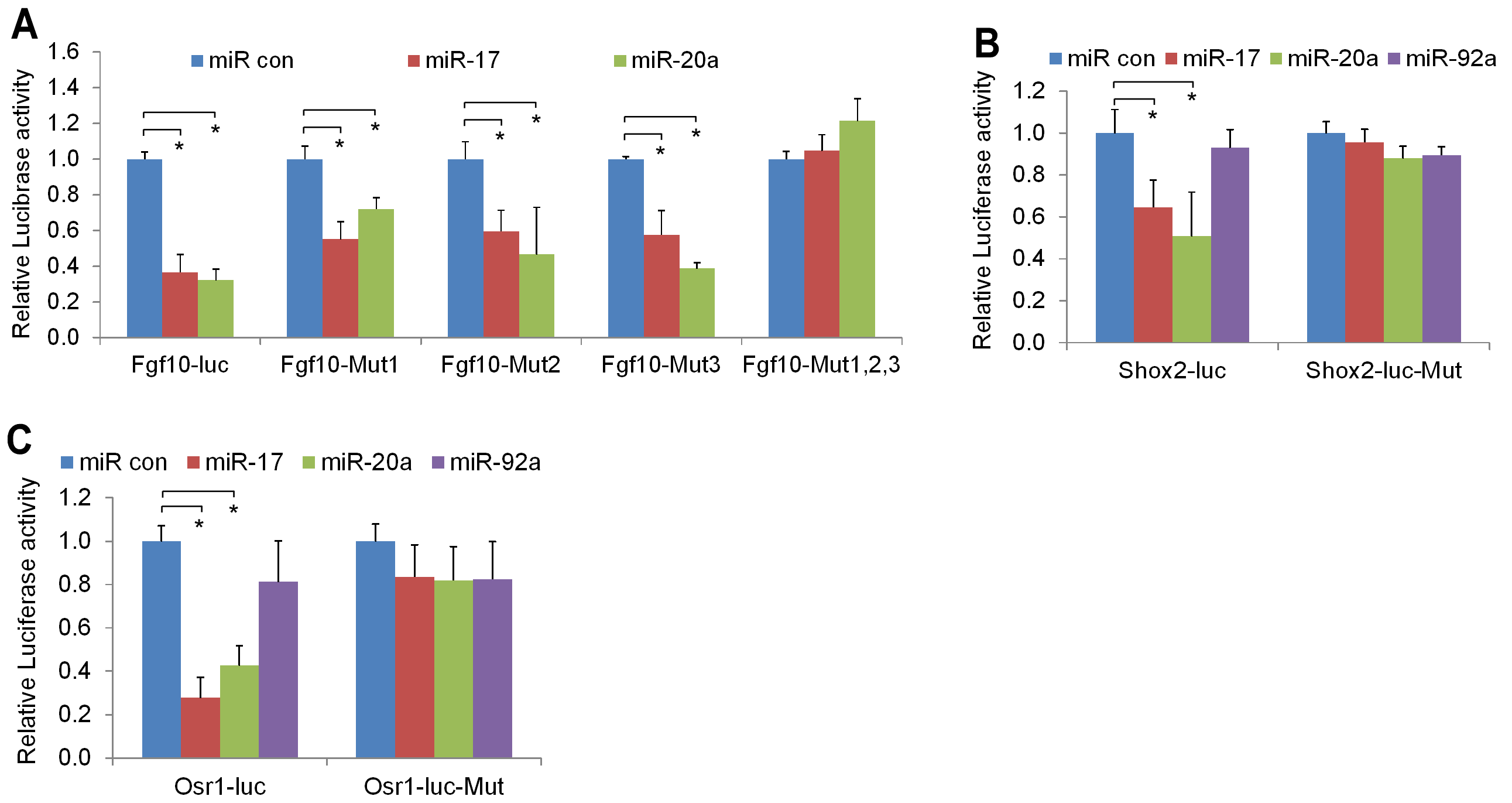

Supplement: Figure S7 — mir-17-92 directly regulates Shox2, fgf10 and osr1. (A–C) Luciferase reporter assays with reporters and miRs as labeled. Mean±s.e.m., * indicates statistically significant difference, Student's t-test (P<0.05). (TIF) [file pgen.1003785.s007.tif]

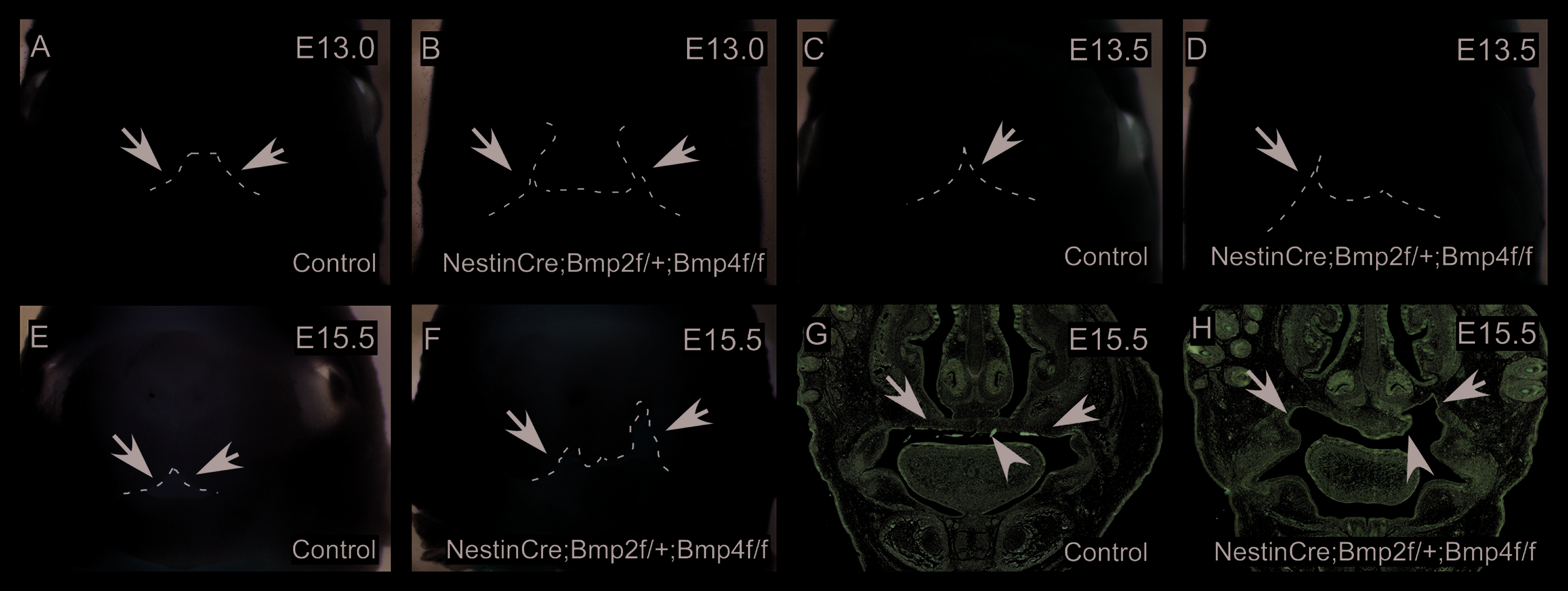

Supplement: Figure S8 — Cleft lip and palate in Bmp CKO mutants. Genotypes and stages of embryos (A–F) and hematoxylin-eosin (HE) staining sections (G–H) are as labeled. Dashed lines show outline of midface, arrows designate fusion or clefting, arrowheads designate palate. (TIF) [file pgen.1003785.s008.tif]

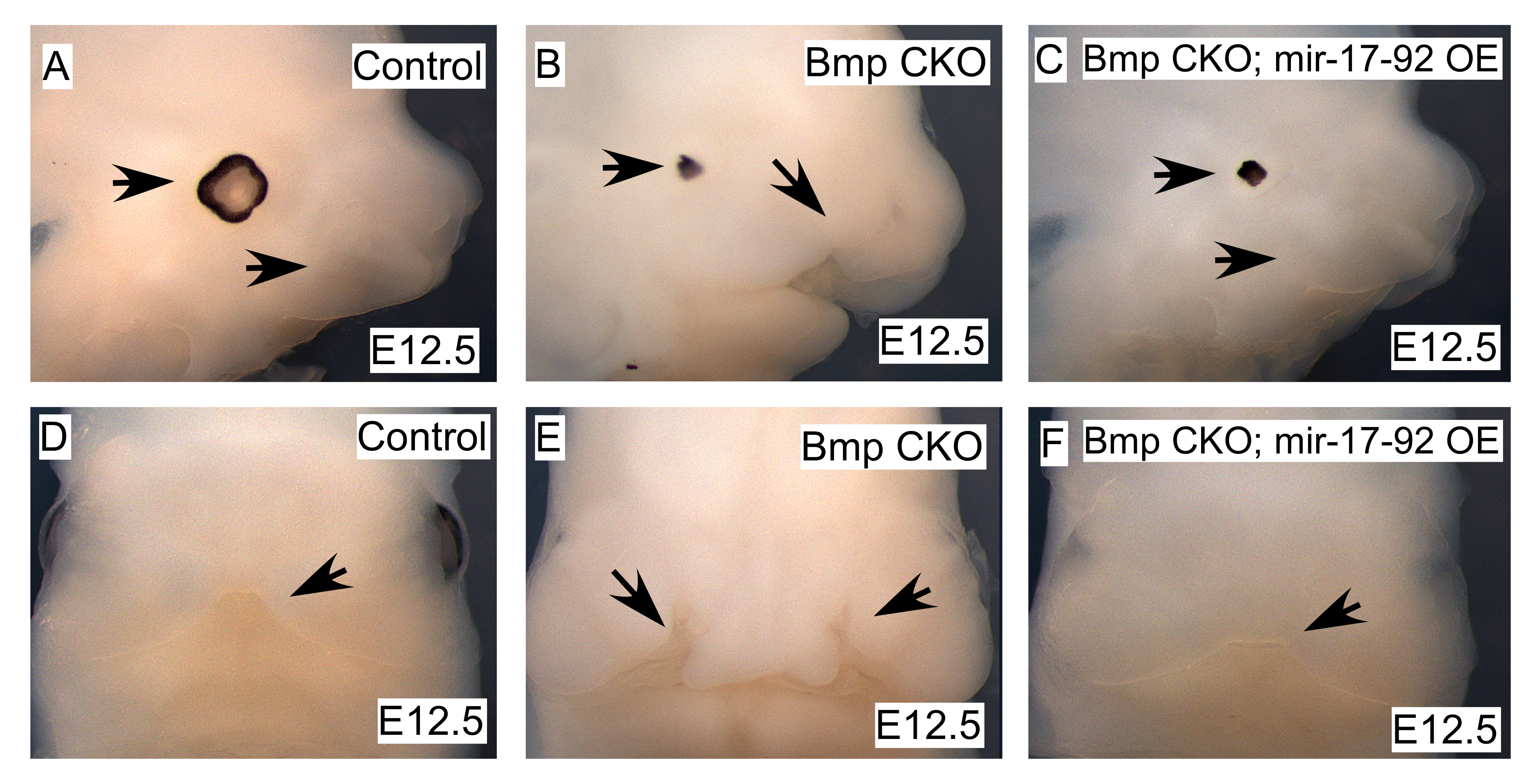

Supplement: Figure S9 — miR-17-92 overexpression rescues the orofacial cleft in Bmp CKO mutants. Embryos with the designated genotypes and designated stages are shown in side views (A–C) and frontal views (D–F). A NestinCre, Bmp4 flox/flox, Bmp7 flox /+ (Bmp CKO) mutant embryo (B, E) had severe bi-lateral cleft, which was observed in in a control (A, C) and a NestinCre, Bmp4 flox/flox, Bmp7 flox /+, miR-17-92OE embryos (Bmp CKO, miR-17-92 OE) (C,F). However, miR-17-92 overexpression did not rescue the eye defect in a Bmp CKO mutant (C). Black arrows designate and orofacial structures. (TIF) [file pgen.1003785.s009.tif]

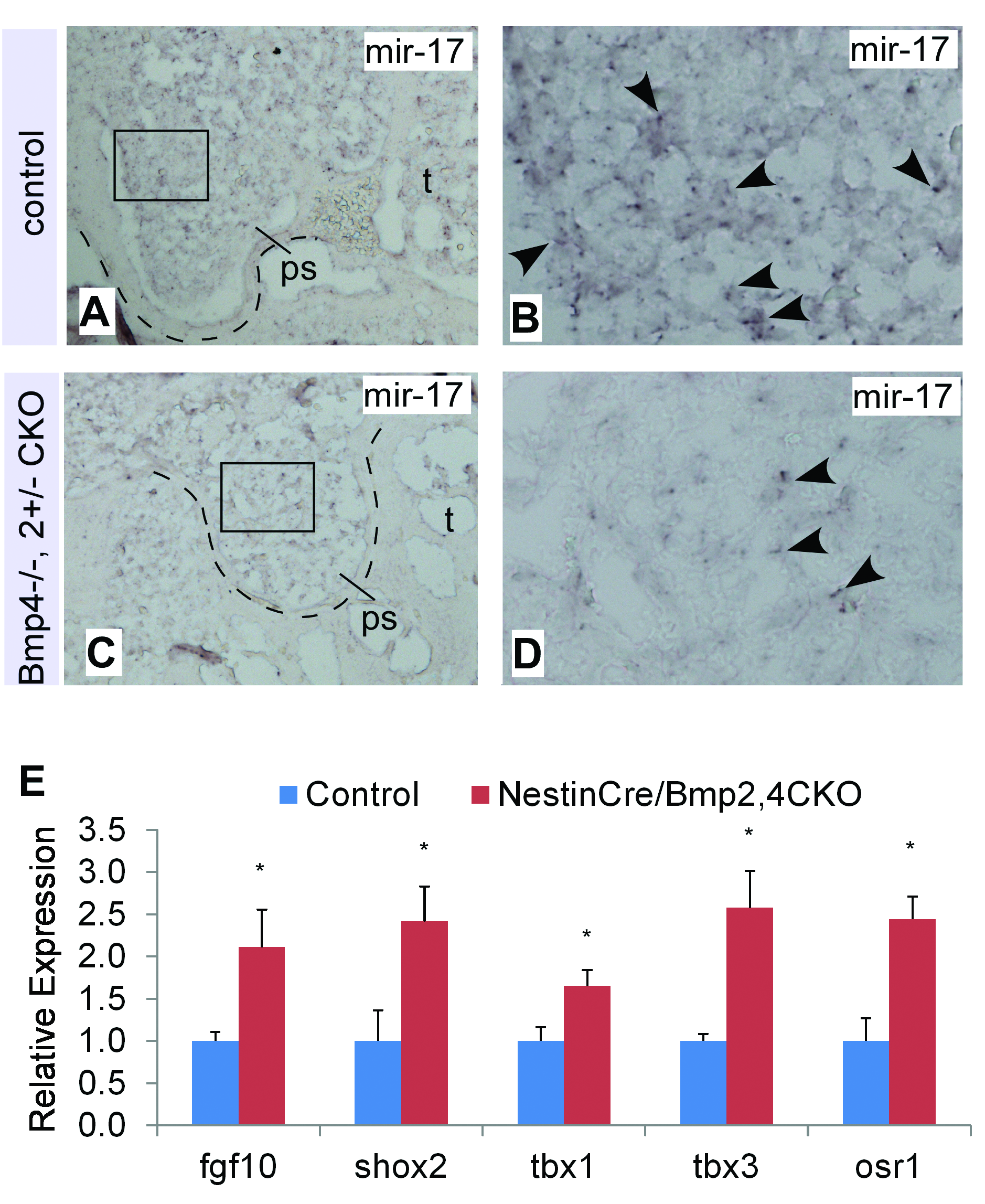

Supplement: Figure S10 — miR-17-92 is a downstream of Bmp signaling. (A–D) In situ analysis of mature miR-17 indicated that mir-17 was dramatically reduced in a NestinCre, Bmp4 flox/flox, Bmp2 flox /+ (Bmp4 −/−, Bmp4 +/− CKO) mutant (C–D) compared to a control (A–B). Boxed areas in A and C are correspondingly shown at higher magnification in B and D. Black arrows designate signals. (E) qRT-PCR data indicate that loss of Bmp signals results in elevation of miR-17-92 target genes including Fgf10, Shox2, Tbx1, Tbx3 and Osr1. Mean±s.e.m., * indicates statistically significant difference, Student's t-test (P<0.05). (TIF) [file pgen.1003785.s010.tif]

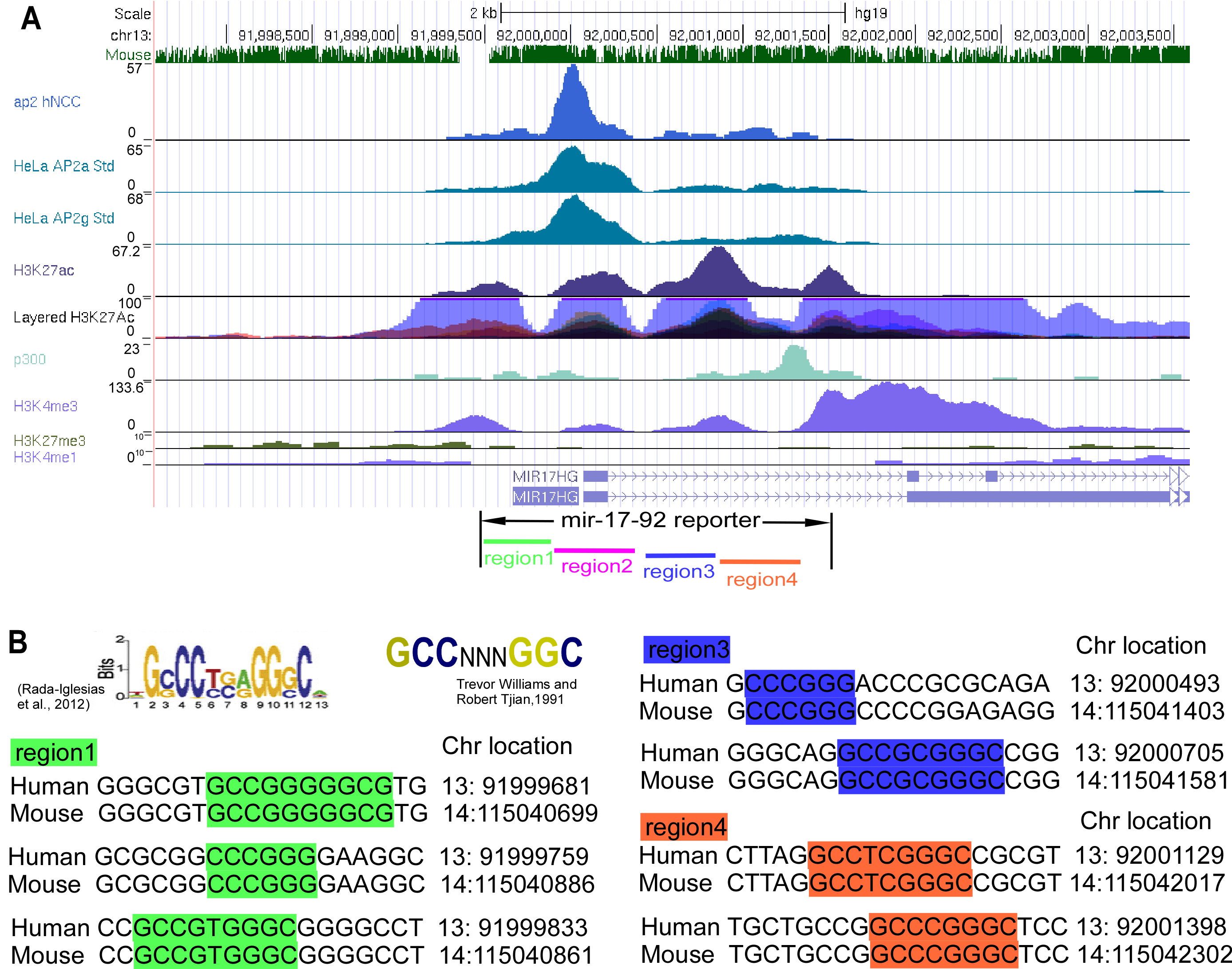

Supplement: Figure S11 — AP-2α directly regulates the miR-17-92 complex. (A) AP-2α and histone modification markers ChIP-seq data in cultured human neural crest and Hela cells. (B) Sequence alignment of AP-2α binding region-1, 3 and 4 in miR-17-92. Region 1 contains three potential binding sites, region 3 and 4 contain two. Region two was shown in Figure 4. (TIF) [file pgen.1003785.s011.tif]

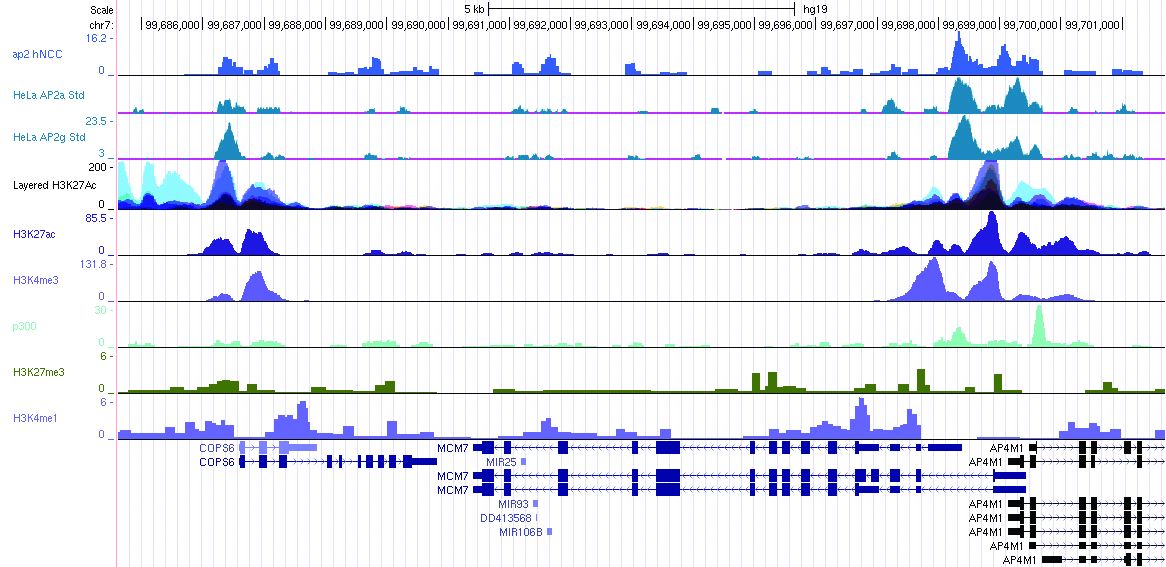

Supplement: Figure S12 — The miR-106b-25 complex is a potential AP-2α target, which is suggested by AP-2α and histone modification markers ChIP-seq data in cultured human neural crest and Hela cells. (TIF) [file pgen.1003785.s012.tif]

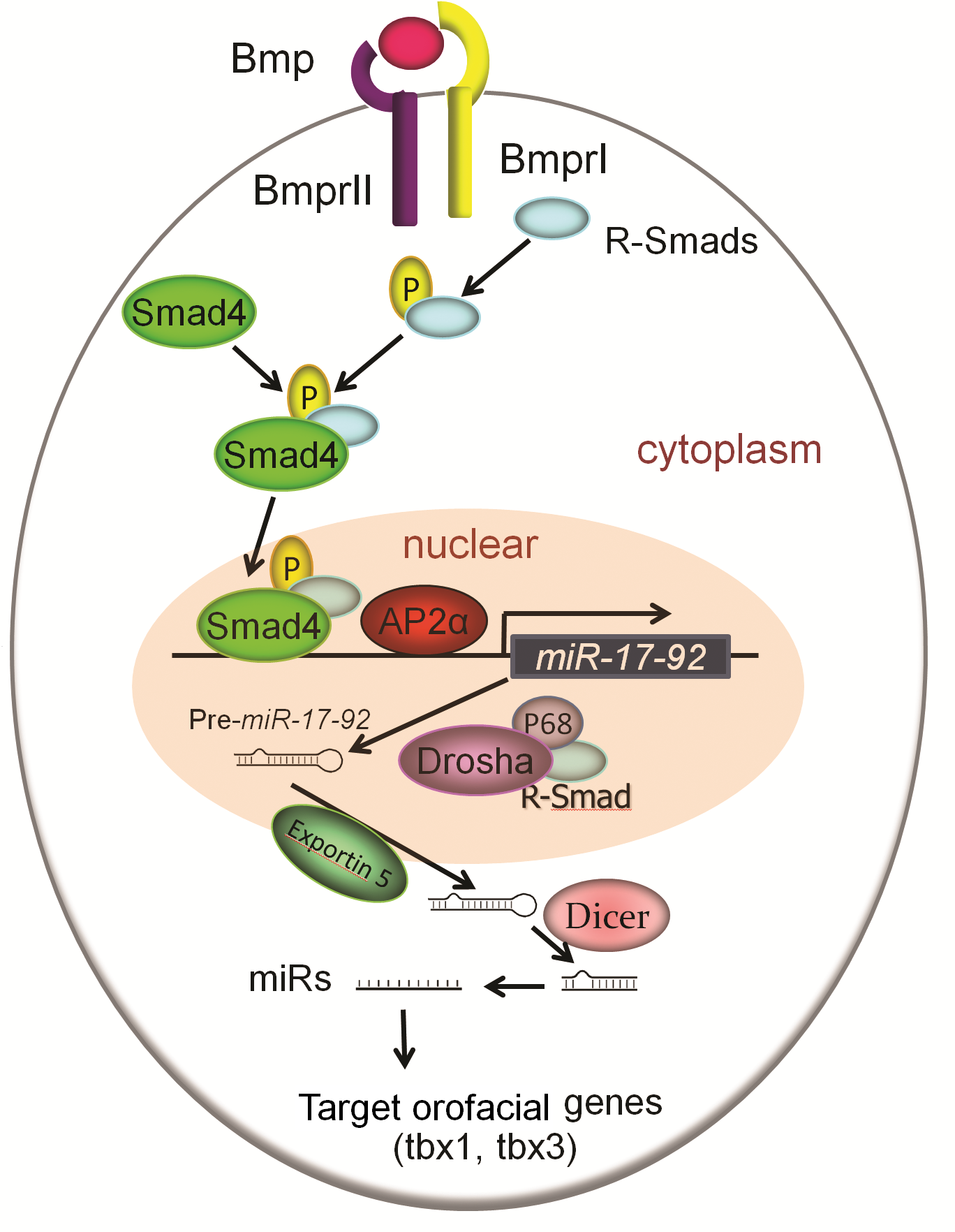

Supplement: Figure S13 — A model for the Bmp/AP-2α-miR-17-92-Tbx pathway during orofacial development. (TIF) [file pgen.1003785.s013.tif]

**Table S1. Summary of phenotypes of *miR-17-92* single and *miR-17-92;miR- 106b-25* compound mutant embryos.**

**
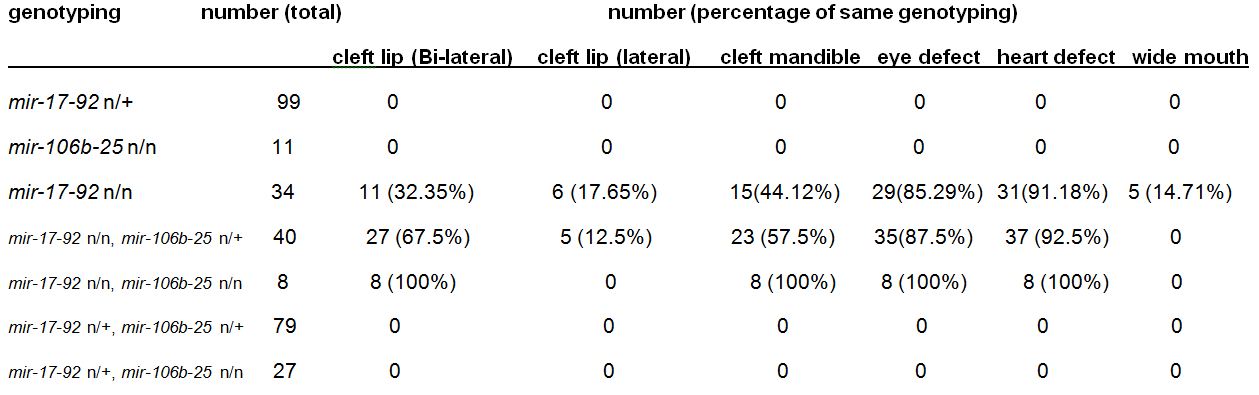
**

Supplement: Table S1 — Summary of phenotypes of miR-17-92 single and miR-17-92;miR- 106b-25 compound mutant embryos. For cleft lip and palate, the penetrance and severity of the phenotype was more severe in compound mutants for both miR clusters. A “wide mouth” phenotype that was observed in some miR17-92 mutants represents an increased distance between the two frontonasal processes and we believe this is an intermediate phenotype between normal and cleft lip. (DOCX) [file pgen.1003785.s014.docx]
