# Supplementary material for: MicroRNA-17-92, a Direct Ap-2α Transcriptional Target, Modulates T-Box Factor Activity in Orofacial Clefting
Source: PLoS Genet. 2013 Sep 19;9(9):e1003785. doi: 10.1371/journal.pgen.1003785 (PMC3777996; doi:10.1371/journal.pgen.1003785)
Supplement: Table S2 — Phenotype summary of embryos with different genotypes. At embryonic day 10.5 to 12.5 (E10.5–E12.5), all Nestin Cre, Bmp4 flox/flox, Bmp7 flox/+ mutant embryos (Nestin Cre, B4 f/f, B7f/+) have severe bi-lateral cleft lip and heart defect. In most case, miR-17-92 over expression rescue cleft lip and heart defect caused by Nestin Cre, B4 f/f, B7f/+ but not eye defect. 83.33% (5 out of 6) Nestin Cre, Bmp4 flox/flox, Bmp7 flox/+, miR-17-92-OE (Nestin Cre, B4 f/f, B7f/+, miR-OE) mutant embryo were fully rescued and 16.67% (1 out of 6) had bi-lateral cleft lip and heart defect. Two Nestin Cre, B4 f/f, B7f/+ mutants died at E12.0 likely due to severe heart defect and 7 embryos were not able to genotyped due to early embryonic lethal at E9.5. * miR-17-92 over expression rescued cleft lip (compared to Bmp CKO, CHI-TEST, p<0.01). (DOCX) [file pgen.1003785.s015.docx]

**Table S2. Phenotype summary of embryos with different genotypes.**

**genotyping number (total) number (percentage of same genotyping)**

**cleft lip (Bi-lateral) cleft lip (lateral) eye defect heart defect___**

*No Cre controls* 29 0 0 0 0

*Nestin Cre*, *B4 f/f, B7f*/+ 6 6 (100%) 0 6 (100%) 6 (100%)

**Nestin Cre*, *B4 f/f, B7f*/+, *miR-*OE 6 1 (16.67%) 0 6 (100%) 1 (16.67%)

*Nestin Cre*, *B4 f/+, B7f*/+ 23 0 0 0 0

Unknown 7 embryonic lethal at E9.5
